# Microporous zirconium-coated titanium surfaces for dental implant application: Surface characterization, bioactivity and effect on the oral biofilm formation

**DOI:** 10.1007/s10856-025-06985-1

**Published:** 2025-12-12

**Authors:** Yumi C. Del Rey, Rubens F. Albuquerque-Junior, Ana Paula Ramos, Bárbara Araújo dos Reis, Leandro Fernandes, Luis G. Vaz, Cássio do Nascimento

**Affiliations:** 1 Department of Dental Materials and Prosthodontics, School of Dentistry of Ribeirão Preto, São Paulo University (USP), Ribeirão Preto, SP Brazil; 2 Department of Chemistry, Ribeirão Preto College of Philosophy Sciences and Letters, São Paulo University (USP), Ribeirão Preto, SP Brazil; 3 Department of Dental Materials and Prosthodontics, School of Dentistry, São Paulo State University (UNESP), Araraquara, SP Brazil

## Abstract

Implantable materials based on titanium have been widely used in medical and dental care due to favorable mechanical properties and biocompatibility. However, when compared to ceramics, titanium surfaces are more susceptible to bacterial adhesion and colonization by periodontopathogenic species. Peri-implant infections are the major cause of implant failure and development of implantable materials with surface properties effective in minimizing bacterial colonization is still a challenge. Plasma electrolytic oxidation (PEO) provides porous ceramic coatings of high durability and stability on titanium. Incorporating metals with antimicrobial effects may contribute to minimizing microorganism adhesion on titanium. This study developed a novel zirconium-coated titanium surface using PEO and compared the composition of oral biofilm with machined or double acid etched surfaces in situ. SEM analysis showed pores and micro-pores, respectively for PEO and zirconium coatings, with surface roughness lower than acid etching while maintaining moderate levels (Ra/Sa: 1.0-2.0 µm). Chemical composition analysis showed predomination of TiO_2_ on the most superficial layer of all groups with phosphorous and calcium incorporated into PEO coatings; calcium was replaced by a relevant amount of zirconium after anodization. Both PEO and zirconium coatings presented reduced values of surface free energy and less wettability than control and etched surfaces. DNA Checkerboard hybridization analysis showed that zirconium coatings significantly reduced the total microbial counts on the formed biofilms with lower counts of opportunistic and pathogenic species. In conclusion, PEO and zirconium coatings have substantially modified the microbial colonization pattern of biofilms, with preferential colonization by commensal Streptococci on zirconium surfaces.

**Graphical Abstract:**

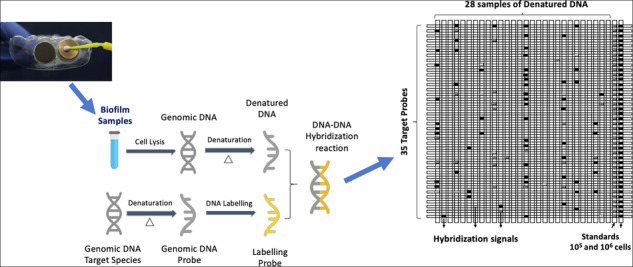

## Introduction

Periimplant-related infections are attributed to the oral biofilm accumulation around the dental implants [1, 2]. The complex structure of this dental devices - including gaps and hollow spaces between its components – associated to the extremely roughness of the external active surface - aiming the perfect osseointegration - generate a conducive environment to the microbial migration, adhesion and biofilm formation [3, 4]. This condition is further enhanced with non-adequate oral hygiene support overtime [5].

Mucositis and periimplantitis have been considered to be initiated with the accumulation of oral biofilm followed by microbial dysbiosis, which is associated with an increase in pathogenic species and decrease in commensal bacteria. Dysbiosis is often driven by the lack of biofilm control, followed by infection and inflammation. The host response and additional risk factors as tobacco or previous history of periodontitis also play a significant hole in the etiopathogenesis of the periimplant diseases. The development of a pathogenic biofilm on the implant surfaces may first result in inflammation and the lack of treatment of periimplant mucositis may lead to the bone resorption and implant loss. Meta-analysis and clinical trials studies have revealed that prevalence of both periimplant mucositis and periimplantitis reach values of up to 50% at the subject [6, 7].

Controlling of oral biofilm is essential to avoid the microbiota disruption and progression of disease. In addition to both self-performed and professional biofilm control, providing a dental implant surface capable of avoid or minimize bacterial adhesion may help to reduce colonization and maturation of oral biofilms [5]. Functionalization or modification of dental implant surface have been proposed aiming to promote osseointegration while reducing the bacterial contamination [8, 9]. Several factors are involved in the microbial cell adhesion to surfaces and interfaces, including physico-chemical surface properties and cell surface biochemical components.

Moderate to high rough surfaces are target in the implant surface modification, since they have been associated with stronger bone responses as a consequence of better cell attachment [10]. Investigations reported that higher rough surfaces achieved a significantly higher percentage of bone-implant contact than smooth surfaces. Conversely, roughness has been pointed for a while to be the major responsible for bacterial adhesion and biofilm accumulation on the surfaces of dental implants [11]. In fact, it plays a relevant role in the earlier stages of biofilm formation, since peaks and valleys may act as reservoir for planktonic bacteria favoring their interaction with the closely implant surface. Currently, it is well known that after bacterial approaching, physico-chemical properties such as electrostatic charge, surface free energy and hydrophobic interaction play a central role in the biofilm proliferation and maturation [12]. In this context, the development of dental implant surfaces rough enough to promote bone cells adhesion and not favor the microbial colonization is still a major concern in dentistry.

Several specific topographical and chemical modification have been introduced to modify the surface characteristics of the titanium in order to improve its interactions with bone e microbial cells [13]. Plasma spraying, blasting, acid etching and anodization or a combination of these techniques have been reported as suitable protocols aiming to alter topographical features of metallic implants. Plasma electrolytic oxidation (PEO), a type of anodizing technique using high voltages, is mainly applied to valve metals or alloys, resulting in porous oxidation ceramic coatings [14, 15]. PEO-coated surfaces have been shown to increase bone remodeling while preventing the bacterial contamination. The main target of PEO modification is to provide deposition of Ca and P on a thick and porous oxide layer aiming to enhance its bone bioactive. In addition, this outermost layer may be supplied with other bioactive valve metal, e.g., zirconium (Zr). PEO-coatings followed by anodization with zirconium results in a microporous layer that may be promising in reducing the microbial adhesion on the dental implant surface. A supposedly lower tendency to accumulate biofilm has been reported as an additional advantage of ZrO_2_ surfaces [16, 17]. Furthermore, literature shows that titanium and ceramic substrates suggest to present a selective prevalence of microbial adhesion and colonization [18, 19]; overall, investigations have suggested that roughness is the major issue favoring the microbial adhesion on titanium surfaces while biofilms on the ZrO_2_ surfaces seems to be more impacted by surface free energy. Although literature presents studies in which modified surfaces and coatings have demonstrated ability to prevent microbial adhesion and biofilm formation on dental implant surfaces, some investigations have reported no significant differences [20]. Most of these studies used only mono-species biofilm or a very restricted mixed biofilm including up to 3-5 bacterial species. The research gaps from these contradictory data must be further explored. In particular, the microbial profile of a more representative oral biofilm has not been assessed in literature up to our knowledge. Assessment of the microbial shift in the more complex oral biofilm composition promoted by titanium modification is still needed and could contribute to control and prevent biofilms. Thus, the aims of the present investigation were: (1) to develop and to characterize two experimental PEO-coated titanium surfaces anodized or not with zirconium; (2) to assess the bioactivity of these experimental surfaces; and (3) to investigate the effect of these experimental surfaces on the microbial profile of up to 35 oral species, including bacteria and fungi. We hypothesized that zirconium-coated surfaces would significantly reduce the microbial colonization of oral biofilm while maintaining their bioactivity.

## Material and methods

### Study design

This investigation was carried out in two different parts: (Part 1) functionalization and characterization of titanium surfaces; and (Part 2) microbiological analysis of the biofilm formation on the experimental surfaces. Randomized parallel groups design was used in the first part. The microbiological assay comprised an in situ controlled split-mouth trial with randomization of experimental and control discs at the anterior and posterior region for either right or left sides. Randomization was generated using a table and allocation was hidden in sequentially numbered packs. Discs with no treatment surface were used as control for both surface characterization and microbiological assays. Blinding was possible during the in situ oral biofilm formation as participants were not aware of the discs placement (treatment or control); also, all the outcome measurements and data analysis were blinded.

### Sample size calculation

Sample size estimation was performed using R software [21]. The microbial count parameter was considered as the primary variable for sample size calculation, since it is the most critical variable of the study. The rank eta squared (rank-based ANOVA) was used for the sample size estimation (N) based on the raw data from the pilot study. Considering the following parameters k = 4 (number of groups), f = 0.68 (predicted effect size), 95% CI (0.43–1.00), significance Level=0,05 and power=0.90, sample size resulted in *N* = 9 for all the quantitative analyses. Finally, all the experimental groups presented a total of 15 sample unites (*n* = 15) with the addition of 6 units for the qualitative analyses.

### Participants selection

The in-situ assay was performed in accordance with the Declaration of Helsinki and its amendments, and received approval from the Local Ethics Committee (process number 21353319.4.0000.5419). Written informed consent was obtained from all participants before the start of the study. Nine healthy subjects (five women, four men) aged between 24 and 29 years (mean-age: 25.2 years) were recruited among graduate students of the Ribeirao Preto School of Dentistry. Prior to enrollment, clinical examinations were carried out to ensure that all participants had enough upper teeth to retain an intraoral splint device, and that they had no signs or symptoms of oral pathologies. The exclusion criteria were as follows: (1) presence of gingivitis, periodontitis or active carious lesions, (2) pregnant or lactating women, (3) smokers, (4) systematically at-risk individuals, (5) therapy with antibiotics or medications that could affect the microbiological status in the 3 months prior to enrollment.

### Titanium specimens (sample units)

This study used 60 commercially pure titanium discs (Ti) of grade IV with a diameter of 10 mm and a thickness of 3.5 mm (Intraoss, Itaquaquecetuba, Brazil). 45 discs were exclusively machined for using in this investigation. 15 discs were provided from the industry etched with nitric acid followed by sulfuric acid, according to the protocol used for the Titaoss commercially available implants. The detailed procedures are considered a trade secret and have not been further described by the donor company. The machined discs were randomly enrolled into 3 groups (*n* = 15) according to the following surface treatments: machined control (C1, no surface treatment); microporous Ti (T1, PEO-treated); and microporous zirconium-coated Ti (T2, PEO-treated and anodized with zirconium). Discs previously treated by the industry constituted the commercial control group (C2, double acid-etched).

### Development of experimental surfaces (Part 1)

With exception of discs from the commercial control group (C2), all the 30 machined Ti discs were polished for 1 min with 600 grit sandpaper in an automatic polishing machine to standardize the tested surfaces at baseline prior to the treatments. 15 discs remained without any treatment and were used as machined control (C1). For the experimental treatments T1 and T2, 30 machined Ti discs were chemically treated with Kroll’s reagent (HF:HNO_3_:H_2_O, 1:3:5) for 1 min, according to ASTM E407-99 standard [22] to remove the naturally formed oxide layer (TiO_2_) on the surfaces. The discs were then ultrasonically cleaned with acetone (10 min) (Sigma-Aldrich, St. Louis, MO, USA) and deionized water (10 min), and air dried before surface treatment.

#### Electrochemical treatments

experimental treatments T1 and T2 were carried out in a two-electrode electrochemical cell under constant magnetic stirring and using a DC power supply (N5771A, Agilent Technologies). The Ti disk was used as the anode, while a stainless-steel plate served as the cathode of the system. All chemical reagents were obtained from Sigma-Aldrich. PEO group (T1) was performed in an electrolyte containing calcium acetate (C₄H₆CaO₄, 62 g/L) and disodium beta-glycerophosphate (C_3_H_7_Na_2_O_6_P) at a current density of 0.707 A/cm² and 300 V during 1 min. Microporous Zr-coated Ti samples (group T2) were obtained by further anodizing half of PEO-treated discs. After ultrasonic cleaning in deionized water (10 min), the PEO-treated discs were anodized in an electrolyte of zirconium oxychloride (Cl_2_OZr, 6 g/L) at a current density of 0.02 A/cm² and 25 V for 10 min. After treatments, the samples from T1 and T2 were ultrasonically cleaned with deionized water (10 min), dried at 40 °C for 1 h and stored in a vacuum desiccator.

### Surface characterization (Part 1)

#### Surface roughness

linear surface roughness (Ra) and average roughness (Sa) of the specimens (N = 9) were measured without contact and observed with a 3D laser scanning confocal microscope (Olympus LEXT OLS4000, Tokyo, Japan) to avoid damage to the studied surfaces. Linear roughness (Ra) was analyzed along three arbitrary radial lines with a cut-off of 80 µm and an evaluation length of 2.57 mm. For average roughness (Sa), three arbitrary rectangular areas were delineated (2.57 mm × 2.59 mm) and measured with a cut-off of 250 µm. The mean value of the three measurements was considered as Sa and Ra values of the respective sample.

#### Morphology and chemical composition

the surface morphology and chemical composition of control and experimental samples (*N* = 3) were investigated by scanning electron microscopy (SEM; EVO 50, Carl Zeiss, Cambridge, England) and energy dispersive X-ray (EDX; 500 Digital Processing, IXRF Systems, Houston, Texas, USA) analysis, respectively. To assess the homogeneity of the Zr-coated surfaces (T2), compositional maps were generated by EDX. The atomic concentration (at.%) of the elements in the top layer ( < 5 mm) was investigated by using high-resolution X-ray photoelectron spectroscopy (XPS; UNI-SPECS UHV System, Berlin, Germany) at a base pressure of 5×10^-7 ^Pa. The Al Kα line was used as the radiation source (hν = 1254.6 eV) and the pass energy of the analyzer was set to 10 eV.

#### Crystalline structure

X-ray diffraction (XRD) analysis was performed to identify the phase composition and crystalline structure of the samples (N = 3). Diffraction patterns were recorded over a 2θ range of 10°–80° with a step size of 0.02° using a Bruker-AXS D5005 diffractometer (Siemens, Hamburg, Germany) equipped with a nickel monochromator filter and Cu Kα radiation (λ = 1.54 Å). The crystalline phases were identified by comparison with the Joint Committee of Powder Diffraction Standards (JCPDS) reference values [23].

#### Wettability and surface free energy

wettability (*N* = 9) was assessed by the sessile drop method using a goniometer (OCA 20-DataPhysics Instruments GmbH, Filderstadt, Germany) by measuring the contact angle (θ) (CA) against distilled water. The surface free energy (SFE; *N* = 9) was calculated using distilled water, diiodomethane and formamide, according to Owens-Wendt equation: γL(1+cosθ)=2(γSd γLd)1/2 + 2(γSp γLp)1/2, where S corresponds to the surface area and L corresponds to the fluid tested. The sum of the dispersive (γ^d^) and polar (γ^p^) components of the tested surface resulted in the total surface free energy (γs).

### Surface bioactivity

Rapid apatite precipitation in a biomimetic environment is considered an indicator of high surface bioactivity. In this study, the bioactivity of control and experimental samples was evaluated by immersing the discs for 24 h (37 °C) in simulated body fluid (SBF) that mimics the pH and ionic composition of human blood plasma. The formation of apatite and/or its precursors on the surface was examined using MEV after gold-sputtering the samples at 40 mA for 150 s (Bal-Tec, SCD-050 Sputter Coater, Los Angeles, CA, USA). The calcium/phosphorus atomic ration (Ca/P) of the surface was determined by EDX (500 Digital Processing, IXRF Systems).

### Microbiological analysis - in situ oral biofilm formation (Part 2)

#### Splint preparation and discs assignment

**Fig. 1:**
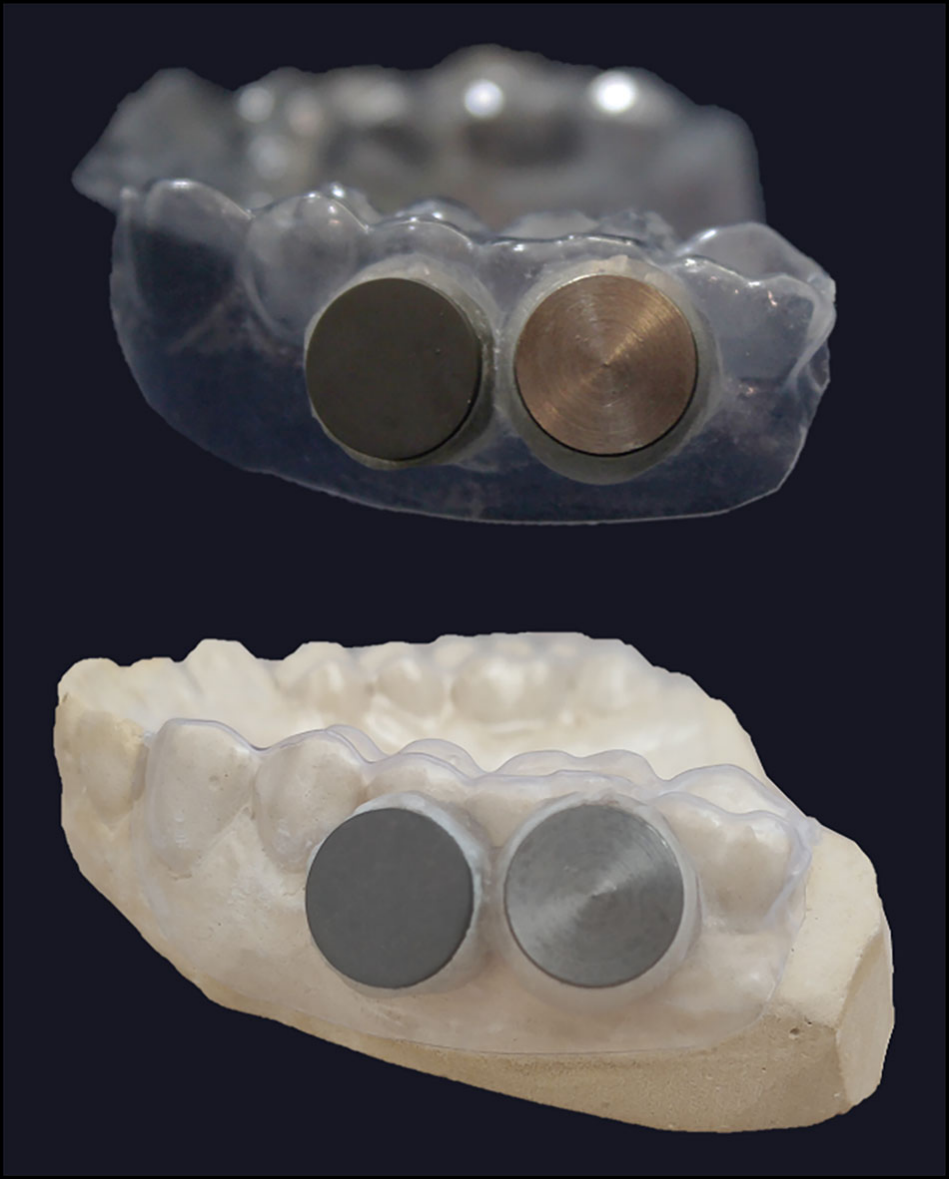
Intraoral splint for in situ contamination test of the Ti discs (top) and their adaptation to the maxillary gypsum cast (bottom). Four discs (one per treatment group) were randomly attached to the premolar or molar regions of the splint

Upper intraoral splints were fabricated to allow in situ contamination of Ti discs and further analysis of the oral biofilm formation (Fig. 1). Briefly, thin acetate plates (0.3 mm) were heated and pressed under vacuum (Plastvac P7, Bio-Art) against the maxillary gypsum casts obtained from the participants. Four Ti discs (C1, C2, T1 and T2) were randomly attached to the premolar or molar areas of the splint using a transparent self-curing acrylic resin. Participants were instructed to wear the splint for two consecutive days (48 h), except while eating or brushing teeth, when the splint was immersed in a 0.9% NaCl solution to avoid dehydration and loss of microbial cell viability. After 48 h, the splints were removed and the oral biofilm formed on the surface of the discs was collected using a sterile regular microbrush (Microbrush International Ltd. Clogherane). All the biofilm samples were placed in individual 1.5 mL microtubes containing 150 µL TE buffer (10 mM Tris-HCl, 1 mM EDTA, pH 7.6) followed by addition of 150 µL NaOH 0.5 M and stored at -20 °C until laboratorial processing using the Checkerboard DNA-DNA hybridization technique.

#### Microbial detection and quantification

A total of 35 microbial species were used as target for detection and quantitation in the oral biofilms formed on the discs. Species included early colonizers and pathogenic microorganisms. They are displayed in Table 1 and were grouped in microbial complexes according to Socransky et al. [24]. The Checkerboard DNA-DNA hybridization method was performed as previously described by our group [25]. Briefly, each biofilm sample (genomic DNA corresponding to an unknown number of microbial cells) was applied to a nylon membrane (Hybond N + , Amersham Biosciences, UK) using the individual slots of a MiniSlot device (Immunetics, Boston, MA, USA). Two slots were used to apply two control samples containing a mixture of genomic DNA corresponding to either 10^5^ cells or 10^6^ cells of all target species. Labeled genomic probes of the 35 target species were individually applied to the slots of the device using a precision pipette, creating a “checkerboard” pattern of sample and probe. After washing the membrane to remove non-specific binding signals, the hybridization signals were detected by chemiluminescence using Gene Images CDP-Star reagent (GE Healthcare, UK). The hybridization signals were transferred to a ECL Hyperfilm-MP (GE Healthcare) and analyzed using CLIQS software (Core Laboratory Image Quantification Software, TotalLab). To estimate the number of microbial cells in the samples examined, the signals emitted by the samples for each target species were compared with the control lanes. The total number of microorganisms and the individual quantification of each target species in the biofilm formed were determined. Data of microbial colonization were also provided by Shannon alfa-diversity index.

**Table 1:** Target species for detection and quantification by Checkerboard DNA-DNA hybridization

Species	ATCC N°	Species	ATCC N°
***Red complex***		***Fungi***	
*Porphyromonas gingivalis*	33277	*Candida dubliniensis*	7984
*Treponema denticola*	35405	*Candida tropicalis*	66029
*Tannerella forsythia*	43037		
		***Other species***	
***Orange complex***		*Bacteroides fragillis*	25285
*Campylobacter rectus*	33238	*Enterococcus faecalis*	700802
*Campylobacter gracilis*	33236	*Escherichia coli*	25927
*Fusobacterium nucleatum*	25586	*Klebsiella pneumoniae*	BAA-1705
*Prevotella intermedia*	25611	*Lactobacillus acidophilus*	4356
*Prevotella nigrescens*	25261	*Lactobacillus casei*	334
*Parvimonas micra*	33270	*Pseudomonas putida*	700007
		*Pseudomonas aeruginosa*	15442
***Yellow complex***		*Peptostreptococcus anaerobius*	49031
*Streptococcus gordonii*	35105	*Prevotella melaninogenica*	25845
*Streptococcus mitis*	49456	*Porphyromonas endodontalis*	35406
*Streptococcus sanguinis*	*10556*	*Streptococcus mutans*	700610
		*Streptococcus parasanguinis*	903
***Purple complex***		*Streptococcus salivarius*	9759
*Veillonella parvula*	10790	*Streptococcus pneumoniae*	6303
		*Streptococcus gallolyticus*	43143
***Green complex***		*Staphylococcus aureus*	25923
*Aggregatibacter actinomycetemcomitans a*	29523	*Solobacterium moorei*	CCUG39336
*Capnocytophaga gingivalis*	33624		

Bacterial strains were displayed into different microbial complexes according to Socransky’s classification (1998); unclassified species were grouped as “other species” or “fungi”. ATCC American Type Culture Collection, Rockville, MD, USA

### Data analysis

Quantitative data were subjected to normal distribution and homogeneity of variances analyses. Roughness, wettability and surface energy were analyzed using ANOVA followed by Tukey multiple comparisons of means. Total microbial counts were compared using Brunner-Langer nonparametric analysis in factorial experiments (ATS: ANOVA-Type Statistics and WTS: Wald-Type Statistics with multiple comparisons adjusted by Bonferroni). Individual microbial counts between groups were compared using the generalized estimating equations (GEE) method. All statistical analyses were performed using R software [21] with a significance level of 5%.

## Results

### Surface characterization

#### Surface roughness

**Fig. 2:**
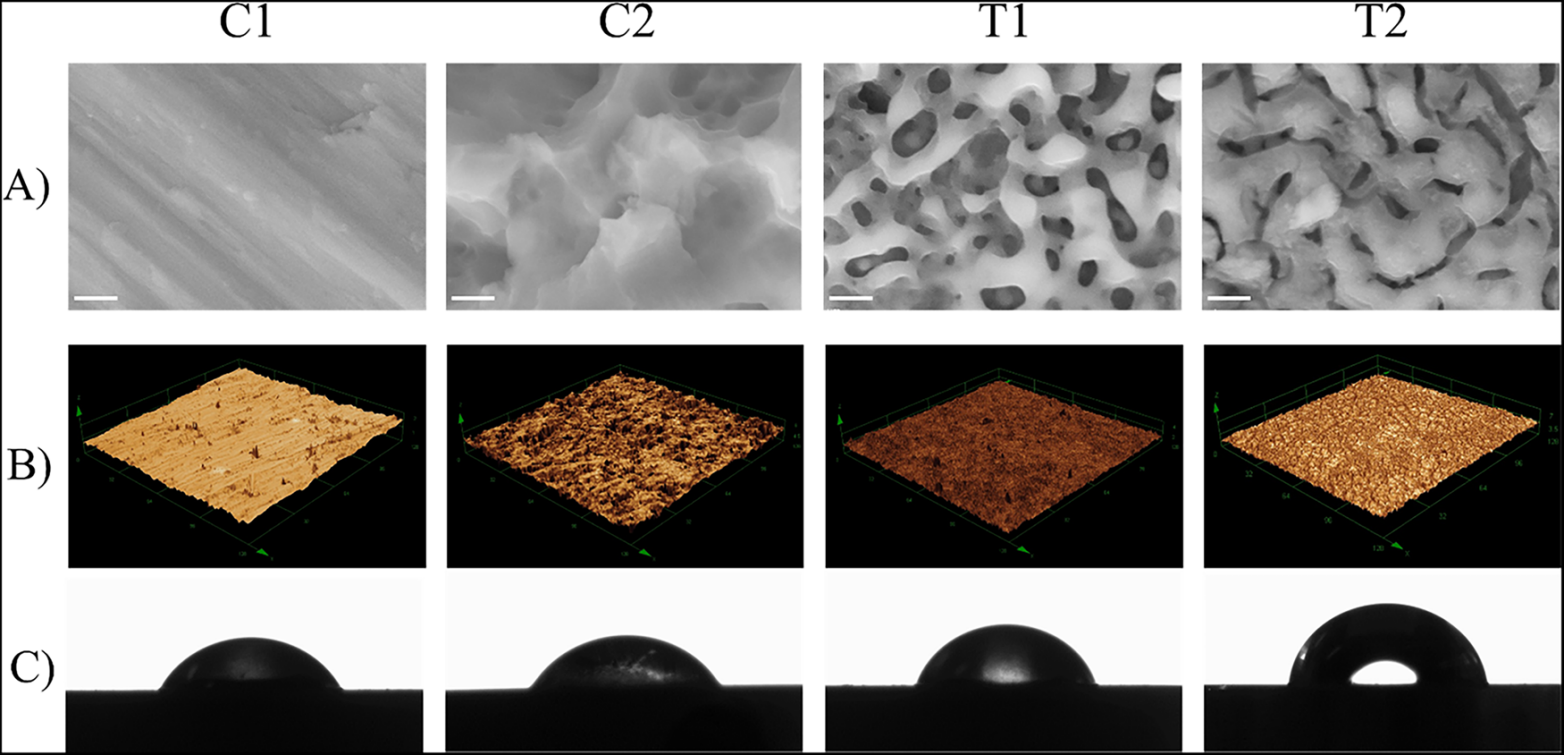
**A** Scanning electron micrographs of control and experimental Ti surfaces; **B** Three-dimensional reconstructions of surface topography obtained by confocal laser microscopy; **C** Representative images of contact angle measurements between the tested surfaces and distilled water. Scale bar: 1 μm

the topography of the control and experimental surfaces was analyzed using linear (Ra) and average roughness (Sa) measurements and 3D reconstructions of the surfaces (Table 2, Fig. 2A). The machined control (C1) surface had the lowest Ra and Sa values (*P* < 0.05) and was classified as a smooth surface (Ra/Sa < 1 µm). PEO treatment (T1) and Zr-coated (T2) resulted in increased roughness (Sa and Ra) compared to the machined substrate (C1); both experimental surfaces were considered moderately rough (Ra/Sa: 1.0–2.0 µm) with no differences between them. Commercial Ti (C2) exhibited the highest Sa and Ra values but was also considered moderately rough (*P* = 1.14×10^-10^). Accordingly, 3D reconstructions of surface topography showed higher roughness for C2 followed by T1 and T2. Machined control (C1) showed relatively smooth topography.

**Table 2:** Linear roughness (R_a_), average roughness (S_a_), surface free energy (γ_s_) and their polar (γ_s_^p^) and dispersive (γ_s_^d^) components, and water contact angle (CA) for control and experimental Ti surfaces

Group	Ra (µm)	Sa (µm)	γ_s_ (mJ m^−2^)	γ_s_^p^ (mJ m^−^^2^)	γ_s_^d^ (mJ m^−^^2^)	CA (^o^)
C1	0.84 ± 0.18 ^A^	1.27 ± 0.16 ^A^	49.59 ± 2.63 ^A^	24.06 ± 3.55 ^A^	25.36 ± 3.27 ^A^	53.44 ± 5.20 ^A^
C2	1.92 ± 0.25^B^	2.38 ± 0.12^B^	50.50 ± 2.29 ^A^	30.66 ± 4.27^B^	20.01 ± 4.44^B,C^	47.44 ± 5.48 ^A^
T1	1.27 ± 0.12 ^C^	1.65 ± 0.10 ^C^	37.00 ± 2.58^B^	18.65 ± 3.13 ^C^	18.18 ± 3.55^B^	63.61 ± 5.89^B^
T2	1.48 ± 0.15 ^C^	1.76 ± 0.17 ^C^	35.62 ± 1.84^B^	13.56 ± 3.59^D^	23.56 ± 3.93 ^A,C^	72.93 ± 5.02 ^C^

Different letters indicate significant differences between groups (P = 1.52 × 10^-31^; Tukey test). C1 machined Ti, C2 commercial double acid-etched Ti, T1 PEO-treated Ti, T2 PEO-treated and Zr-coated Ti

#### Morphology and chemical composition

The SEM images illustrated in Fig. 2B show the formation of a patterned microporous coating on the surfaces of T1 and T2 discs as a result of PEO treatment. The zirconium homogeneously covered the formed micropores in group T2, slightly reducing the porosity of the surface while maintaining the “volcano-like” structure. C1 exhibited parallel linear scratches on the surface, consistent with the machining process. In C2, peaks and valleys were observed, which were caused by the acid etching of the surface.

**Fig. 3:**
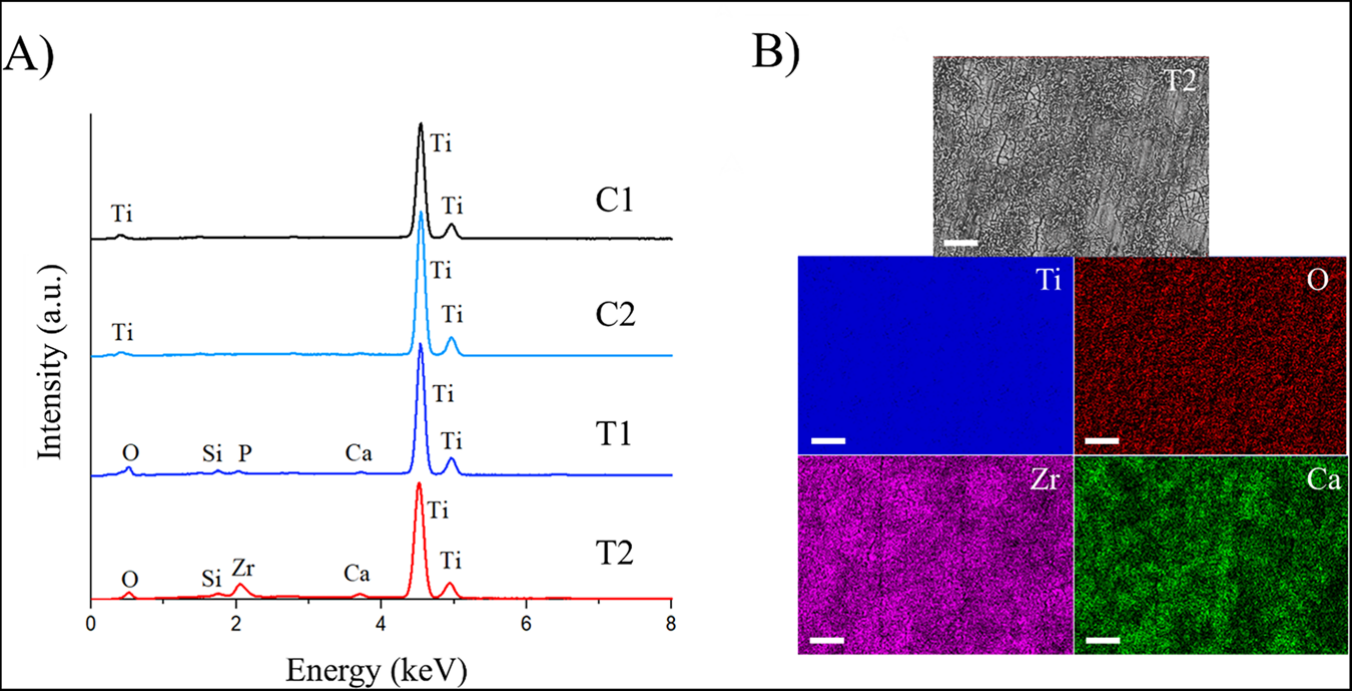
**A** EDX analysis of the chemical composition of control and experimental surfaces; **B** EDX mapping of the distribution of Ti. O. Zr and Ca on microporous Zr-coated Ti surfaces

EDX analysis was performed to characterize the chemical composition of the surface (Table 3, Fig. 3). Only Ti was found in the control groups (C1 and C2), probably due to the limited thickness (3–7 nm) of the passive TiO_2_ layer formed spontaneously on the Ti surfaces. In this study, the PEO treatment was not specifically designed to change the chemical composition of the Ti surface; however, the elements phosphorus (P) and calcium (Ca) from the PEO electrolyte were still detected on the T1 samples. For the Zr-coated samples, compositional maps showed the successful and uniform deposition of Zr on the surfaces, as well as the presence of Ti, O and Ca.

**Table 3:** Atomic concentration (at.%) of the elements Ti, O, Ca, P and Zr on control and experimental surfaces assessed by Energy Dispersive X-ray Analysis (EDX) and X-Ray Photoelectron Spectroscopy (XPS)

	EDX (at.%)					XPS (at.%)*				
Group	Ti	O	Ca	P	Zr	Ti	O	Ca	P	Zr
C1	100	-	-	-	-	31.7	66.5	1.8	-	-
C2	100	-	-	-	-	31.6	66.2	2.3	-	-
T1	54.73	43.82	0.47	0.99	-	2.1	73.7	3.2	21.0	-
T2	55.38	40.15	1.14	-	3.33	2.1	68.9	-	7.0	22.0

*At.% obtained from high resolution spectra at the most superficial layer ( < 5 nm) of the tested surfaces. C1 machined Ti, C2 commercial double acid-etched Ti, T1 PEO-treated Ti, T2 PEO-treated and Zr-coated Ti

**Fig. 4:**
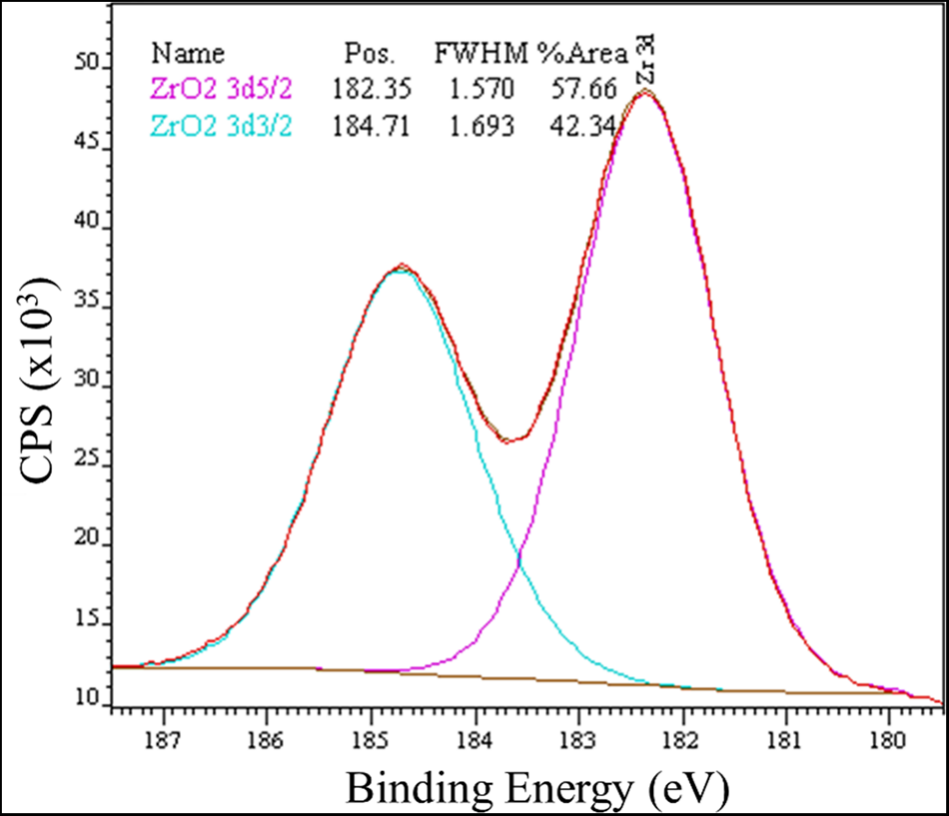
Deconvoluted Zr 3 d spin-orbit XPS spectrum (3d5/2 and 3d3/2) obtained for the Zr-coated PEO-treated sample. The position of the Zr 3d5/2 peak at 182.4 eV corresponds to the monoclinic ZrO_2_ phase

The atomic concentration of the chemical elements in the outermost layer of the Ti samples (<5 nm) was further quantified by XPS for more accurate chemical characterization (Table 3, Fig. 4). The XPS analysis revealed the same elemental composition for the T1 surfaces as the EDX analysis. The control groups (C1 and C2) had Ti and O at a Ti/O ratio of 0.48, suggestive of TiO_2_, along with traces of Ca, likely due to contamination during sample manipulation. The Zr-coated surfaces (T2) had a high concentration of Zr and O, indicating the formation of ZrO_2_.

#### Crystalline structure

**Fig. 5:**
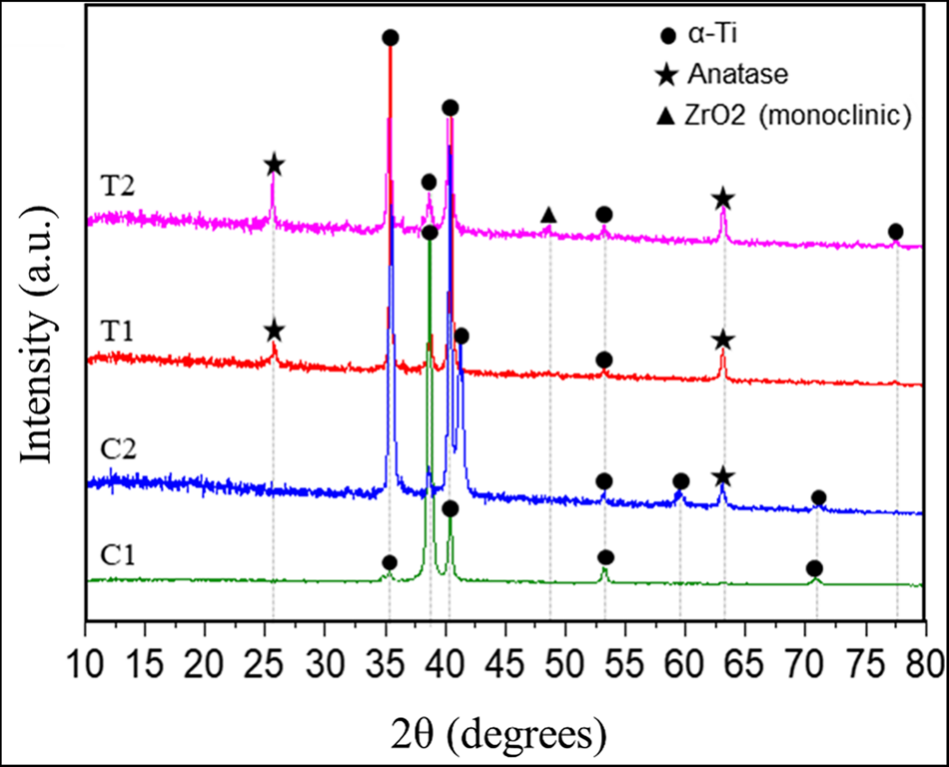
X-ray diffraction patterns showing the phase composition of the control and experimental surfaces

The crystalline composition of the control and experimental groups was evaluated by XRD analysis. The XRD patterns of the control and experimental samples and the corresponding JCPDS references are shown in Fig. 5. The diffractogram of the machined control surface (C1) exhibited exclusively reflections associated with α-titanium, indicating that the machining process did not induce the formation of detectable crystalline oxide phases. In contrast, samples C2, T1, and T2 displayed additional diffraction peaks consistent with the formation of crystalline TiO_2_ in the anatase phase, with characteristic reflections observed at 2θ = 25.62° and 63.03°. Furthermore, the T2 sample exhibited an additional diffraction peak at 2θ = 48.66°, corresponding to the monoclinic ZrO_2_ crystal phase. This finding indicates the incorporation of zirconium to the titanium surface, resulting in the formation of a mixed titanium-zirconium oxide surface layer. ZrO_2_ formation was also confirmed by XPS, a more sensitive tool for analyzing the chemical state of Zr cations on the surface coatings. The deconvoluted XPS spectra for Zr 3 d showed peaks at 182.35 V and 184.71 V, corresponding to the Zr 3 d3/2 and Zr 3d5/2 components, respectively. The observed binding energy values for the Zr 3d components are consistent with the formation of monoclinic ZrO_2_, thus confirming the successful incorporation of a crystalline ceramic layer on T2.

#### Wettability and surface free energy

The contact angles (CA) and surface free energy (SFE) calculated for each group are displayed in Table 2. Figure 2 shows the CA between the tested surfaces and a drop of distilled water. All tested surfaces were found to be hydrophilic (θ < 90°). No differences in wettability were observed between C1 (CA: 53.44° ± 5.20°) and C2 (CA: 47.44° ± 5.48°), but both control groups showed significantly higher hydrophilicity than T1 (CA: 63.61° ± 5.89°) and T2 (CA: 72.93° ± 5.02°) (*P* = 1.53×10^-5^). The Zr-coated samples (T2) were the most hydrophobic surfaces, with CA values significantly higher than those of the other groups (*P* = 8.04×10^-9^). The SFEs for the experimental groups (T1: 37.00 ± 2.58; T2: 35.62 ± 1.84) were significantly lower compared to the control groups (C1:49.59 ± 2.63; C2: 50.50 ± 2.29) but no significant differences were observed between C1 and C2 or between T1 and T2 (*P* = 0.1113). Regarding the polar component, all groups differed significantly from each other (C2 > C1 > T1 > T2; *P* = 3.52×10^-7^), with the experimental groups showing the highest values. The PEO treatment significantly decreased the dispersive component of SFE compared to the machined substrate (C1: 25.36 ± 3.27; T1:18.18 ± 3.55;) (*P* = 1.29×10^-31^); however, subsequent Zr coating of the PEO-treated samples significantly increased the dispersive component of SFE (T2: 23.56 ± 3.93), resulting in values similar to C1 (*P* = 0.4371). Commercially treated Ti showed lower dispersive component values (C2: 20.01 ± 4.44) compared to C1 (*P* = 0.0016), but no significant differences were observed between C2 and T1, and C2 and T2.

### Surface bioactivity

**Fig. 6:**
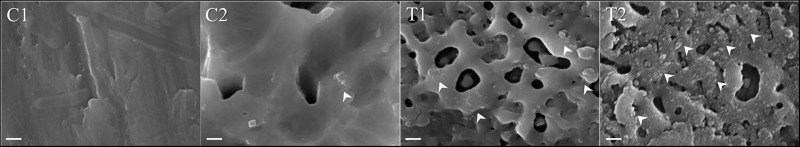
Scanning electron micrographs of control and experimental surfaces after 24 h immersion in simulated body fluid at 37 °C. White arrows indicate calcium phosphate aggregates (Scale bar: 1 μm)

The apatite forming ability of the surfaces was evaluated after a very short exposure (24 h) under biomimetic conditions to determine which surface treatments would first stimulate the nucleation and growth of apatite crystals and/or their precursors. The results are shown in Fig. 6. After exposure to SBF, virtually no precipitating particles were present on C1 and C2. A larger but relatively sparse deposition of calcium phosphate aggregates was observed on T1. The Zr-coated surfaces (T2) exhibited significantly increased bioactivity compared to the other groups, as indicated by the substantial formation of calcium phosphate clusters on the surface, mainly along the walls of the micropores. These spherical clusters, known as Posner clusters, are considered precursor building blocks for the formation of hydroxyapatite (HA). EDX analysis revealed a Ca/P atomic ratio of 1.17 for T2, consistent with the formation of brushite (CaHPO_4_.2H_2_O) and octo-calcium phosphate (Ca_8_(HPO_4_)2(PO_4_)4.5H_2_O), which are transitional phases preceding the nucleation of HA. The Ca/P ratios for C1, C2 and T1 were 0.09, 0.16 and 0.60, respectively.

### Oral biofilm formation

#### Total microbial count

**Fig. 7:**
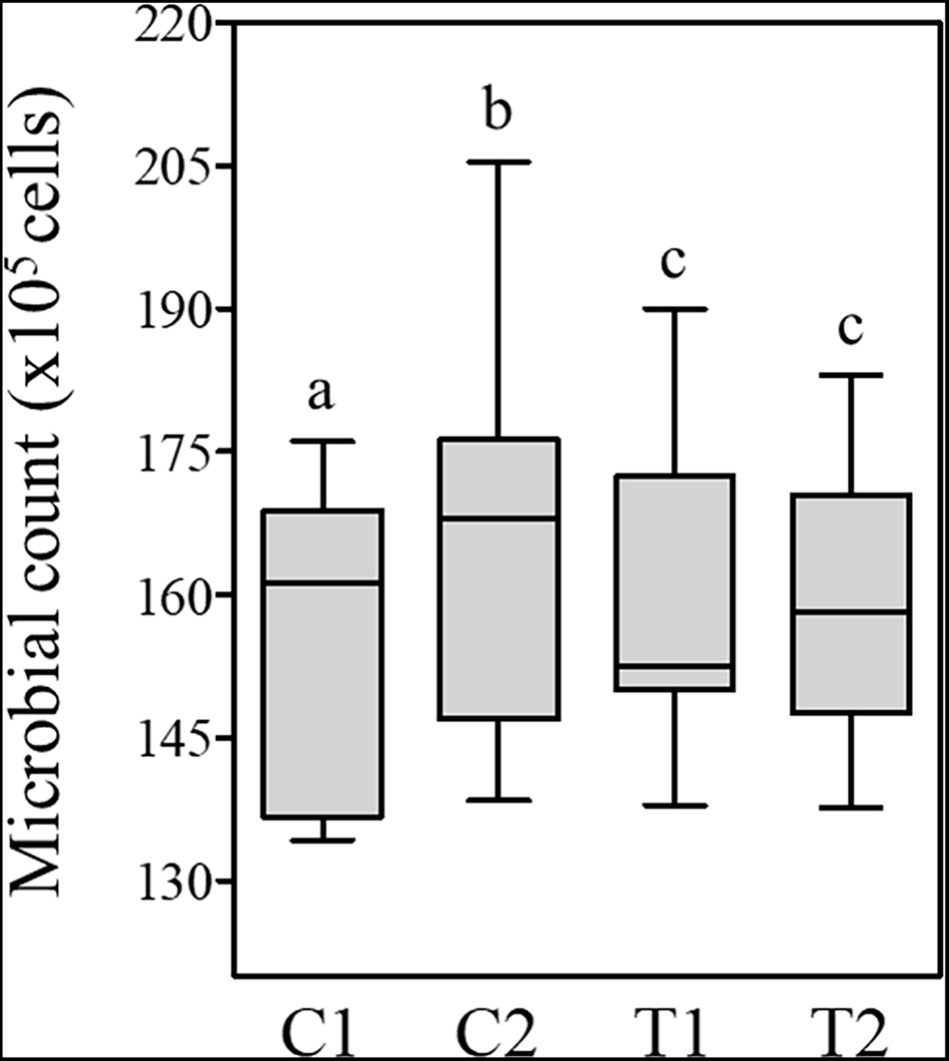
Total microbial counts (median, interquartile range, maximum and minimum) of the in situ grown biofilms assessed by Checkerboard DNA-DNA hybridization. Different letters indicate significant differences between groups (ATS: *P* = 1.14×10_-10_ and WTS: *P* = 1.52 × 10^-31^)

Figure 7 shows the median, interquartile range, and maximum and minimum values of the total microbial counts of the biofilms formed on the Ti samples. The highest median was observed for the commercial treatment (C2), followed by the machined control (C1), and the lowest microbial counts were observed on the experimental surfaces (T1 and T2). Multiple comparisons differences sought by ATS and WTS (C1 – C2: p = 1.43x10^-5^; C1-T1: p = 0.26; C1-T2: 0.075; C2-T1: *p* = 0.00089; C2-T2: *p* = 0.00057; T1-T2: *p* = 0.63). No significant differences were observed between T1 and T2 (*P* = 0.3685).

#### Individual microbial quantification

**Fig. 8:**
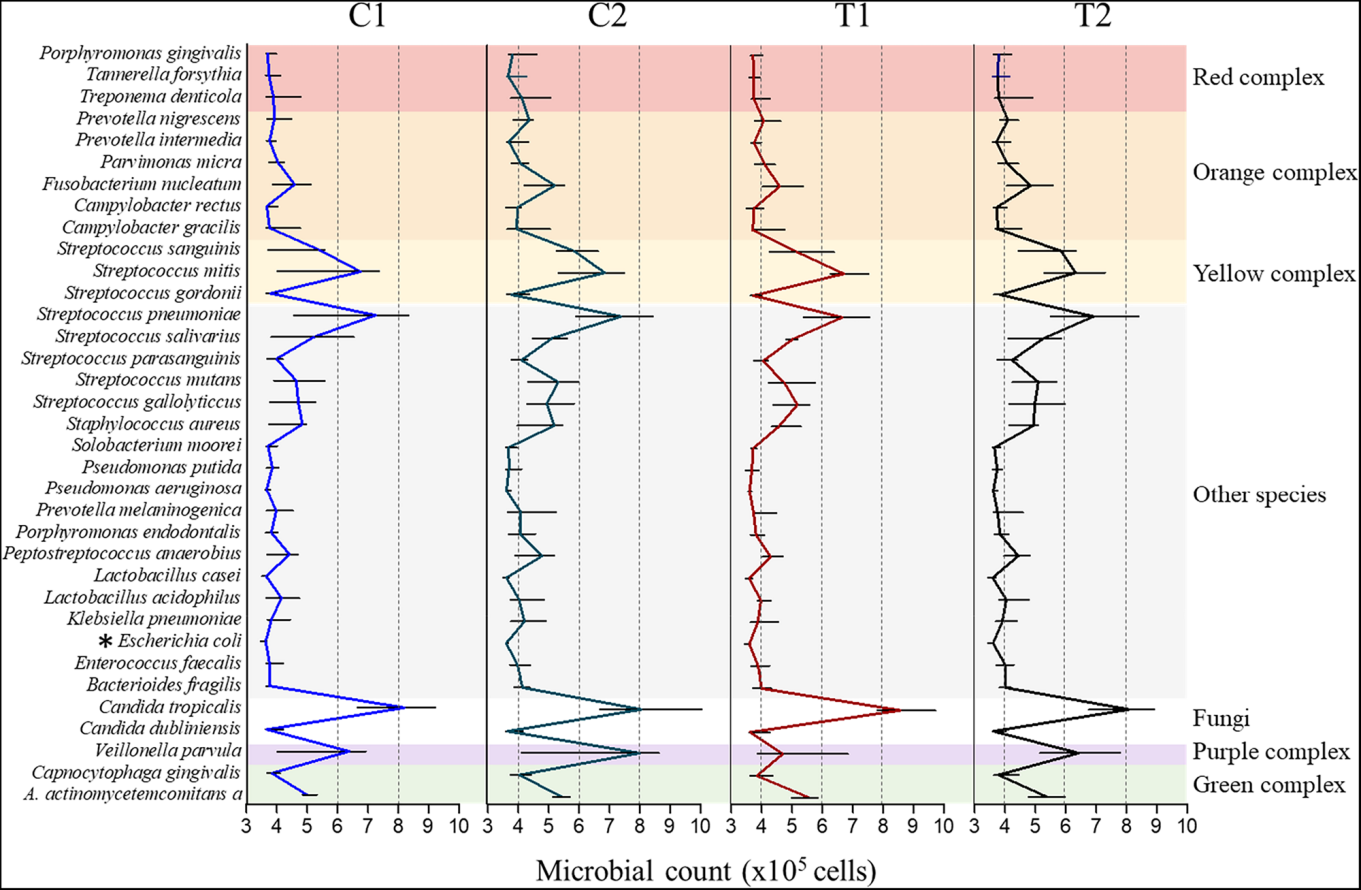
Individual microbial counts (median and interquartile range) of the 35 target species obtained by DNA-DNA checkerboard hybridization. *Escherichia coli did not differ between groups (Generalized Estimated Equations; *P* < 2 × 10^-16^)

The individual counts of the 35 target microbial species are illustrated in Fig. 8 and grouped into complexes according to Socransky’s classification [24]. All the tested microorganisms, including pathogens and commensals, were identified colonizing the biofilms formed on the different Ti surfaces. Statistical analysis showed a significant effect for the “treatment” factor, the “microbial species” factor, and for the “treatment x microbial species” interaction (*P* = 8.14×10^-10^). Of all target microorganisms, only *Escherichia coli* did not differ significantly between treatments (*P* = 0.9541). Shannon alfa-diversity index was high for all tested groups with no significant differences (C1: 3.33; C2: 3.45; T1: 3.27 and T2: 3.11; *P* = 0.3975), which means that all substrates harbor high diversity of microorganisms with no dominant species.

Overall, different microbial colonization patterns were identified on the different Ti substrates. A total of 20 of the 35 microbial species examined were found in high numbers on C2 compared to T1 and T2 (*P* < 2×10^-16^). These include several species from the orange complex (*Prevotella nigrescens, Parvimonas micra, Fusobacterium nucleaum, Campylobacter rectus, Campylobacter gracilis*), the purple complex (*Veillonella parvula*) and strictly anaerobic bacteria (*Bacterioides fragilis, Capnocytophaga gingivalis, Peptostreptococcus anaerobios, Porphyromonas endodontalis, Prevotella melaninogenica*). Also, some opportunistic microorganisms (*Staphylococcus aureus, Candida dubliniensis* and *Klebsiella pneumoniae*) were also found in higher concentrations on C2 compared to the experimental treatments. Conversely, several streptococci (*Streptococcus sanguinis, Streptococcus salivarius, Streptococcus parasanguinis, Streptococcus gordonii*), which are considered early colonizers of the oral biofilms, adhered preferentially to Zr-coated surfaces than to commercially treated Ti.

The control groups also differed significantly in terms of biofilm composition. The vast majority of the microorganisms studied were identified at higher concentrations on C2 compared with C1. A few exceptions were some opportunistic bacteria (*K. pneumoniae, Candida tropicalis* and *C. dubliniensis*), species from the green complex (*Aggregatibacter actinomycetemcomitans* and *C. gingivalis*), two streptococci (*S. mitis* and *S. gallolyticcus*) and two aerobic bacteria (*Solobacterium moorei* and *Pseudomonas aeruginosa*). Regarding the experimental surfaces, Zr-coated treatment (T2) significantly changed the microbial profile of the formed biofilms. In the T1 discs, some late and intermediate colonizers were found in higher abundance (*T. denticola, P. intermedia* and *C. rectus*), while T2 showed high number of other pathogens (T*. forsythia, P. gingivalis, F. nucleatum* and *C. gracillis*). As for the early colonizers of the oral biofilm, a clear preferential colonization of streptococci (except *S. gallolyticcus* and *S. mitis*) was observed in Zr-coated surfaces compared to T1. No clear colonization trend was observed for the bacteria of the red complex, and both the control and experimental groups showed relatively low numbers of these species.

## Discussion

Controlling of oral biofilms on the dental implant surfaces has currently been one of the main targets in dentistry studies [26, 27]. Some modified titanium surfaces including topographical and/or chemical alterations have been proposed to reduce the microbial colonization and proliferation while maintaining the potential of osseointegration [28, 29]. However, most of these studies have only used mono-species biofilm or very restricted mixed biofilms (with few species belonging to same genus or different genera) to assess bacterial contamination. In the present investigation, we conducted an in situ model assay to capture the complex interaction between up to 35 oral species colonizing the biofilm formed on PEO-coated or PEO-zirconium-coated surfaces. These species included bacteria and fungi commonly found in the oral cavity. A complete topographical surface characterization and the bioactivity of formed layers were also provided.

Our results from the anodic oxidation have produced different topographies and chemical compositions of surface oxides on titanium discs. SEM analysis showed pores and micro-pores, respectively for PEO-coating and Zirconium-coating in the surface oxides of titanium. These findings are in accordance with the most common characteristics for PEO oxide layers, which normally exhibits a porous texture resulting in moderate to high roughness [30, 31]. The sizes of pores are not uniform and depend on the electrochemical parameters used during anodic oxidation procedure. Electrical potential and concentration of electrolyte may significantly influence both uniformity and size of pores [32]. Through the process of proposed anodization of zirconium, the pores were reduced in size, leading to the creation of micro-pores. As a result, we obtained a surface uniformly covered by a ceramic surface of zirconium. These findings are important to prevent the microbial attachment. Surfaces with larger dimension porous provide greater available contact area and sheltering for microbials, thereby resulting in an enhanced microbial adhesion [33]. XPS analysis of the chemical composition showed that the predominant surface oxide formed on the most superficial layer of all groups is TiO_2_, as expected. Additional phosphorous and calcium were incorporated into the oxide on PEO-coating discs on the top of the layer as well as at the bottom, as observed by EDX analysis. Differently, in the zirconium-coated discs, calcium was replaced by a relevant amount of zirconium at the most superficial layers. The carbon contaminations on the different oxides in our tested groups were quite similar and reached values about 15-20%, which is not representative to influence the results obtained [34, 35]. Probably, the effectiveness of anodization process must have contributed to reduce the contaminants in the outer layer of discs. XRD analysis revealed the presence of anatase peaks on the titanium surfaces from the C2, T1, and T2 groups, confirming that the applied surface treatments promoted surface oxidation and the formation of a crystalline titanium dioxide layer. Additionally, XRD showed that the T2 treatment not only oxidized the titanium surface but also facilitated the incorporation of zirconium into the surface layer, resulting in the formation of monoclinic ZrO₂. This phase is commonly associated with enhanced osteoblastic activity and improved antibacterial performance [36].

PEO treatment and Zirconium-coatings presented surface roughness significantly lower than acid etching discs while maintaining the moderate levels (Ra/Sa: 1.0–2.0 µm). These results are interesting, because the experimental surfaces were considered smoother than etched commercial surfaces, which is better to preventing microbial attachment and further biofilm colonization and not compromise the osseointegration process. It is believed that the ideal implant surface for bone remodeling and osseointegration can be achieved with Ra/Sa values between 1.0 and 2.0 µm [37, 38]. Other relevant findings of the present investigation corroborate to the potential anti-microbial effect of experimental surfaces; both PEO-coated and Zirconium-coated surfaces presented reduced values of surface free energy and higher contact angle (less wettability) when compared to control and etched surfaces. This means the experimental surfaces in the present study are not conducive to microbial attachment and suggest that surfaces may difficult or inhibit the biofilm growth, since several studies reported that increased surface energy and wettability led to increased bacterial adhesion and biofilm formation on titanium or ceramic surfaces [39–41]. However, there is no consensus on the literature regarding the impact of surface free energy and wettability on microbial adhesion and biofilm growth of substrates. Other investigations showed different conclusions, in which bacterial adhesion was enhanced by hydrophobic surfaces [42, 43].

These contradictory data may be explained, in part, by the multifactorial factors involved in the microbial adhesion. We should consider that other relevant factors, as ionic strengths and biological process, may significantly influence the microbial adhesion in different substrates [44]. Microbial cell polarity is mediated by the presence of specific proteins which will influence the electrostatic attraction between oppositely charged ions (substrate-microbial cell). Overall, higher ionic strengths increase microbial adhesion [45]. Other bacterial proteins, adhesins, have a substantial role in the adhesion process. Several bacterial adhesins with individual receptor specificities have been identified in different microorganisms, suggesting a probably specific mechanism for selective microbial adhesion [44, 46]. Investigating the impact of electrostatic charges and bacterial proteins enrolled in the biofilm formation on modified dental implant surfaces are crucial to be addressed in future studies. Finally, all the topographical and chemical composition changes promoted by the PEO and zirconium anodization have not compromised the bioactivity of experimental surfaces. Deposition of calcium phosphate aggregate was observed in higher counts for both treatments, with zirconium-coated surfaces exhibiting enhanced bioactivity as indicated by clusters formation on the surface. Studies involving culture cells or bone remodeling in animal experimental models would also contribute to elucidate these features.

From microbiological analysis, total and individual microbial counts distinctly revealed different results between groups with the change of topography and chemical composition of experimental surfaces. Both PEO-coated and zirconium-coated surfaces showed lower total microbial counts when compared with control and etched surfaces. These data are in line with the physicochemical findings of the present study and seem to corroborate with most of literature. Initially, the reduced dimensions of peaks and valleys of experimental surfaces have difficulted the microbial cells approximation and attachment. Initially, the modification of topography from porous to microporous after zirconium coatings have substantially reduced the microbial counts. Following the biofilm formation, the low free energy and reduced wettability must have negatively influenced the microbial proliferation and biofilm growth. Reduction of roughness, wettability and surface energy implies in less microbial adhesion and biofilm growth [39–41]. Irrespective of reduced counts in both PEO and zirconium coated surfaces, our results found no differences between them. A possible rationale may be the complex physical and chemical molecular interaction between host, microbial cells and different substrates. Differences in the electrostatic attraction mediated by proteins suggest specific ways for selective microbial adhesion. The molecular composition and ionic strengths are essential for bacterial adhesion to the substrate influencing the pattern of microbial colonization. Thus, changes in the biofilm’s protein structure may impact overall biofilm formation, selecting beneficial or pathogenic microorganism [47].

The microbial profile of the 35 target species provided by Checkerboard DNA-DNA hybridization analysis also showed significant differences between tested surfaces. Different microbial profile patterns were observed comparing the different substrates. Both Gram-positive and Gram-negative species can adhere onto titanium and ceramic substrates [48]. However, in the present study, Gram-positive species were more attracted by zirconium surfaces; it seems that topography and physical properties of ceramic surfaces affected their adsorption and adhesion. In vivo studies have also showed this pattern of colonization on ceramic substrates [18, 19]. Notably, most of target species were found in higher counts on etched titanium compared to experimental PEO and Zirconium coatings, including commensals and pathogenic species. In contrast, the zirconium-coated surfaces presented reduced counts of microorganisms and the biofilm composition of was predominantly composed by commensal Streptococci, Gram-positive aerobic microorganisms that are considered early colonizers of the oral biofilms. Literature shows that increased levels of Streptococci may have beneficial effects on the oral health, helping to maintain microbial homeostasis and stability in the long term; they play a role in preventing the colonization of pathogenic bacteria while producing antimicrobial substances (such hydrogen peroxide) and/or competing for nutrients [49]. Also, Streptococci have relevant role in the immune modulation; they can stimulate the expression of antimicrobial peptides (such β-defensins) that preferentially target periodontal pathogenic bacteria [50]. Modulation of the of ecological balance is an important issue, since the oral biofilm dysbiosis with an increase in anaerobic Gram-negative bacteria have been frequently associated with increased biofilm accumulation and disruption on periimplant diseases [51]. An interesting fact observed in our results was that zirconium coating changed the microbial profile of red complex species when compared to PEO coating; *T. denticola* was reduced while *P. gingivalis* and *T. forsythia* were increased. All these species are strongly implicated as playing roles in the etiology and progression of periodontal diseases [52]. Previously, literature demonstrated that *P. gingivalis* play an essential role in synergistic polymicrobial biofilm formation with *T. denticola* stimulating the host immune response and inducing bone loss [53]. Disruption of the cross-feeding between *P. gingivalis* and *T. denticola* in zirconium-coated surfaces may represent a potential breakdown in this pathogenic synergistic pathway. *C. tropicalis* and *C. dubliniensis* were also found in reduced counts on zirconium-coated surfaces, which may be justified by the morphology of these cells, which is large and presents filaments that may have limited their adhesion on the small porous of zirconium experimental surfaces. The shifting in the microbial profiles resulted from the experimental treatments have not modified the microbial diversity of related biofilms. The higher Shannon indexes observed for all substrates indicate a more diverse and potentially healthier biofilm [54]. It was expected, since all the participants enrolled in this study presented good oral health with no signs or symptoms of oral pathologies. Maintaining the biofilm balance with no dominant species is essential to the oral healthy. Noteworthy, the changes in the microbial profiles after experimental treatments suggests that zirconium-modified surfaces may be a choice for individuals with chronic periodontitis or those with high potential to develop periimplant diseases.

The hypothesis tested in this study was confirmed since the results demonstrated that zirconium-coated surfaces significantly reduced the microbial colonization of oral biofilm and exhibited increased bioactivity compared to the other groups. Our study, being of an exploratory nature, provides relevant insights and raises a number of research gaps and opportunities for future investigations. While using an in situ experimental model was representative of the real biofilm formed in the oral cavity and allowed investigating the impact of surface modifications on a large panel of microorganims, our findings cannot be directly translated to the clinical practice. The short exposure time of 48 h may not accurately represent complex mature biofilms or simulate in vivo immune responses. However, by acting as an interim method between clinical trial and basic research, our study provided valuable insights that may significantly improve the efficiency in conducting future clinical researches. Wear resistance is a notable concern on the mechanical and physical features of titanium coatings and should be further investigated. The combined effect of corrosion and frictional wear (tribocorrosion) during prolonged use may lead to gradual delamination of coating, compromising their stability over time. Furthermore, the biocompatibility of proposed coatings is another area of concern; the cellular responses, such as reduced osteoblast adhesion and/or inflammatory reactions, should considered in future investigations using cell culture and animal models. Finally, the absence of differences in the total microbial counts between PEO-coated and zirconium-coated surfaces associated to the quite different pattern of individual microbial colonization for both surfaces should be further investigated considering the potential aspects involved in the molecular interactions. More research will in fact be necessary to overcome our limitations and further elucidate our novel findings. In this context, the present findings could contribute significantly to subsequent preclinical studies and clinical trials.

## Conclusions

In this study, we demonstrated that: (1) Zirconium-coated surfaces exhibited the highest bioactivity; (2) PEO and zirconium coatings have significantly reduced the total microbial counts of formed biofilms with no differences between them; (2) PEO and zirconium coatings presented lower counts of opportunistic and pathogenic species; (3) PEO and zirconium coatings have substantially modified the microbial colonization pattern of biofilms, with preferential colonization by commensal streptococci for zirconium surfaces.
